# Occlusal Overload and Periodontitis: Integrating Mechanisms, Clinical Evidence, and Emerging Perspectives—A Scoping Review

**DOI:** 10.1155/ijod/9936924

**Published:** 2026-03-02

**Authors:** Pietro Leone, Julie Toby Thomas, Timo Sorsa, Mauno Könönen, Sukumaran Anil

**Affiliations:** ^1^ Independent Researcher, SmileLongevityCare360 Centre, Naples, Italy; ^2^ Department of Oral and Maxillofacial Diseases, University of Helsinki and Helsinki University Hospital, Helsinki, Finland, helsinki.fi; ^3^ Department of Periodontics, Saveetha Dental College and Hospitals, Chennai, India, saveetha.com; ^4^ Department of Dental Medicine, Karolinska Institutet, Stockholm, Sweden, ki.se; ^5^ Department of Prosthetic Dentistry, University of Helsinki, Helsinki, Finland, helsinki.fi; ^6^ Faculty of Dentistry, Chulalongkorn University, Pathumwan, Bangkok, Thailand, chula.ac.th; ^7^ Global Research Cell, Dr. D. Y. Patil Dental College and Hospital, Dr. D. Y. Patil Vidyapeeth, Pimpri, Pune, India, dpu.edu.in

**Keywords:** aMMP-8, extracellular matrix degradation, inflammation mediators, occlusal, occlusal adjustment, occlusal loading, periodontal biomarkers, periodontitis, trauma

## Abstract

**Background:**

Occlusal trauma has long been debated as a contributing factor in periodontitis. Excessive occlusal loading acting on inflamed tissues may accelerate periodontal breakdown through inflammatory and proteolytic pathways. This scoping review synthesizes current evidence from clinical, animal, and in vitro studies on the mechanistic and clinical interplay between occlusal trauma and periodontal degradation.

**Methods:**

In accordance with Preferred Reporting Items for Systematic Reviews and Meta‐Analyses extension for scoping reviews (PRISMA‐ScR), PubMed, Web of Science, Scopus, Embase, and Cochrane Library were searched for studies published 2014–2025. Clinical trials, observational studies, animal models, and in‐vitro experiments evaluating occlusal loading in periodontitis were eligible. Study selection followed the PECO framework and targeted inflammatory mediators, aMMP‐8 activity, and clinical periodontal outcomes.

**Results:**

Eighteen studies met the inclusion criteria: eight clinical, seven animal, and three in vitro. Clinical studies have reported that occlusal adjustment, when combined with periodontal therapy, yields greater reductions in probing depth (PD), bleeding on probing (BoP), and pathogenic bacterial load compared to periodontal therapy alone. Animal and in vitro studies consistently demonstrate that mechanical loading amplifies inflammatory cytokine production (e.g., interleukin [IL]‐6 and TNF‐α), activates signaling pathways (inhibitor of kappa B kinase/nuclear factor kappa [IKK/NF‐κB] and yes‐associated protein [YAP]), and suppresses osteogenic differentiation. The release of matrix metalloproteinases (MMPs), particularly MMP‐8, was a recurring feature associated with collagen degradation.

**Conclusions:**

Occlusal trauma functions as a modifying factor that may exacerbate periodontitis progression in susceptible individuals, though it should not be considered an initiating cause of disease. The integration of occlusal assessment, active MMP‐8 (aMMP‐8) biomarker analysis, and targeted therapies may enhance periodontal treatment outcomes. However, the modest effect sizes observed in clinical studies suggest that selective rather than routine occlusal intervention may optimize the benefit‐to‐burden ratio. Further longitudinal and interventional studies are warranted to validate these findings and establish clinical guidelines.

## 1. Introduction

Occlusal trauma is traditionally defined as injury to the tooth’s supporting structures, including the periodontal ligament (PDL), alveolar bone, and cementum, resulting from excessive or abnormal occlusal forces. It is broadly categorized into two types: primary occlusal trauma, which occurs when abnormally high forces (e.g., from bruxism, premature contacts, or ill‐fitting restorations) act on an otherwise healthy periodontium, and secondary occlusal trauma, where even normal occlusal forces adversely affect a periodontium that has been compromised by prior periodontal disease [1, 2]. The 2017 classification system jointly issued by the American Academy of Periodontology and the European Federation of Periodontology (AAP/EFP) recognizes secondary occlusal trauma and bite collapse as defining clinical features of Stage IV periodontitis, reflecting the growing consensus that occlusal dysfunction contributes significantly to disease severity and progression [2, 3].

Despite decades of research, significant gaps persist between mechanistic understanding and clinical decision‐making regarding occlusal trauma in periodontal therapy. Current clinical guidelines lack consensus on the indications, timing, and methods for occlusal assessment and intervention in periodontitis management. This ambiguity risks either over‐treatment with unnecessary occlusal adjustments or under‐treatment by overlooking modifiable biomechanical factors. The absence of standardized diagnostic criteria and the heterogeneity of available evidence further complicate clinical translation. This scoping review is, therefore, timely, as it aims to synthesize the evolving evidence base, clarify the role of occlusal trauma as a modifying factor rather than a causative agent, and identify priorities for future research and guideline development.

Excessive or misdirected occlusal forces can intensify periodontal tissue breakdown by amplifying inflammatory responses and biomechanical stress. Mechanistically, these forces activate intracellular signaling cascades, such as inhibitor of kappa B kinase/nuclear factor kappa (IKK/NF‐κB‐light‐chain‐enhancer of activated B cells), yes‐associated protein (YAP), and interleukin‐6/phosphoinositide 3‐kinase/mitogen‐activated protein kinase (IL‐6/PI3K/MAPK), which promote the release of pro‐inflammatory cytokines (e.g., IL‐1β), enhance osteoclastogenesis via receptor activator of nuclear factor kappa‐B ligand (RANKL), and suppress osteogenic differentiation [4–6]. These molecular pathways collectively trigger the release of proteolytic enzymes, including matrix metalloproteinases (MMPs), such as active MMP‐8 (aMMP‐8), which degrade collagen and extracellular matrix components, thereby accelerating connective tissue destruction and alveolar bone loss [6].

These biomechanical and inflammatory effects often act synergistically with microbial biofilm‐induced damage, contributing to the formation of deeper periodontal pockets, increased clinical attachment loss (CAL), and more rapid bone resorption. Experimental and clinical studies demonstrate that interventions, such as selective grinding, splinting, or coronoplasty, can effectively redistribute occlusal forces, reduce tooth mobility, and improve periodontal healing when used in conjunction with conventional periodontal therapy. For instance, retrospective analyses have shown that patients with untreated occlusal discrepancies exhibit a faster progression of probing depth (PD) and tissue breakdown than those who receive occlusal adjustment. Furthermore, scaling and root planing (SRP) followed by occlusal adjustment significantly reduces collagenase activity in the sulcular fluid of hypermobile teeth with deep pockets, while no such reduction is observed in untreated sites [2].

Advancements in diagnostic modalities, including digital occlusal analysis (e.g., T‐scan) and point‐of‐care molecular profiling, now enable earlier and more precise identification of occlusal discrepancies and active periodontal tissue degradation [7]. Among these, aMMP‐8‐based chairside assays serve as sensitive, noninvasive tools for detecting ongoing collagen breakdown, aiding in assessing treatment efficacy, and guiding personalized therapeutic interventions [8]. Although establishing a definitive causal relationship between occlusal trauma and periodontitis remains challenging, primarily due to ethical limitations on experimental human trials, current evidence strongly supports occlusal trauma as a modifying or exacerbating factor in the progression of periodontal disease.

Given this context, it is hypothesized that occlusal trauma through the induction of microdamage and aseptic inflammation may activate enzymatic cascades that accelerate collagen degradation in periodontal tissues. Although significant progress has been made in elucidating the interplay between biomechanical forces and microbial dysbiosis in periodontitis, the evidence remains heterogeneous and fragmented. This scoping review aims to synthesize findings from the past decade of human clinical research, animal studies, and in vitro models to clarify the diagnostic features, mechanistic pathways, and therapeutic implications of occlusal trauma in periodontal disease. The goal is to determine the extent to which occlusal trauma functions as a cofactor in periodontitis pathogenesis and to highlight emerging strategies for its identification and management in clinical practice.

## 2. Materials and Methods

### 2.1. Study Design and Research Question

This scoping review was conducted following the Preferred Reporting Items for Systematic Reviews and Meta‐Analyses extension for scoping reviews (PRISMA‐ScR, 2020) guidelines. An integrative scoping approach was adopted rather than the stringent protocols typical of systematic reviews to capture the breadth of mechanistic, clinical, and translational perspectives relevant to periodontal pathophysiology.

The research question was structured using the PECO framework:

• Population (P): human patients with periodontitis, animal models with experimentally induced periodontal disease, or in vitro periodontal cell culture models.
• Exposure (E): occlusal trauma or excessive mechanical loading.
• Comparator (C): not mandatory; when available, included healthy controls or models under physiological loading conditions.
• Outcome (O): indicators of periodontal collagen degradation, including clinical, radiographic, biochemical, or histological assessments of inflammatory markers, proteolytic enzyme expression (e.g., MMPs), and their activation products.

The primary aim was to investigate the role of occlusal trauma in promoting collagen degradation and activating tissue‐degrading enzymes in periodontal tissues across clinical studies, animal models, and in vitro experiments published between 2014 and 2024.

### 2.2. Eligibility Criteria

Studies were included if they met following criteria: (1) published in English between January 2014 and December 2024; (2) assessed the relationship between occlusal trauma or overload and periodontal tissue degradation; and (3) reported periodontal clinical parameters—such as bleeding on probing (BoP), gingival index (GI), PD, CAL, radiographic bone loss (RBL) or biological indicators (inflammatory cytokines, bone resorption markers, and matrix degradation enzymes such as MMPs, particularly MMP‐8). Eligible study designs, including clinical controlled trials, longitudinal and cohort studies, case–control studies, relevant case series, animal experimental models, and in vitro cell culture studies. No restrictions were imposed based on study setting or type of outcome measurement, provided outcomes were pertinent to the research question.

Studies were excluded if they met following criteria: (1) published before 2014; (2) focused exclusively on physiological orthodontic forces without reference to traumatic loading or collagen degradation; (3) were narrative reviews, systematic reviews, isolated case reports, or non‐peer‐reviewed materials; or (4) investigated biomarkers without contextual linkage to occlusal trauma or periodontal inflammation.

### 2.3. Search Strategy

Two independent reviewers conducted systematic searches across five electronic databases: PubMed/MEDLINE, Web of Science, Scopus, Embase, and the Cochrane Library. The initial search was performed in July 2024 and updated in December 2024.

The search strategy combined free‐text keywords and Medical Subject Headings (MeSH) using Boolean operators:

• Exposure terms: “occlusal trauma” OR “traumatic occlusion” OR “occlusal overload” OR “excessive occlusal forces.”
• Disease terms: “periodontitis” OR “periodontal.”
• Outcome terms: “collagen degradation” OR “collagen breakdown” OR “extracellular matrix” OR “active MMP‐8” OR “matrix metalloproteinase‐8” OR “neutrophil collagenase” OR “periodontal ligament.”

Reference lists of eligible articles and recent reviews were manually screened to identify additional relevant studies. Conference proceedings and the ClinicalTrials.gov registry were also reviewed, but no additional unpublished data were identified.

### 2.4. Study Selection

Search results were imported into Zotero reference management software. After duplicate removal, titles and abstracts were independently screened by two reviewers. Full‐text articles were retrieved for potentially relevant studies, and disagreements were resolved through discussion or consultation with a third reviewer.

**Figure 1:**
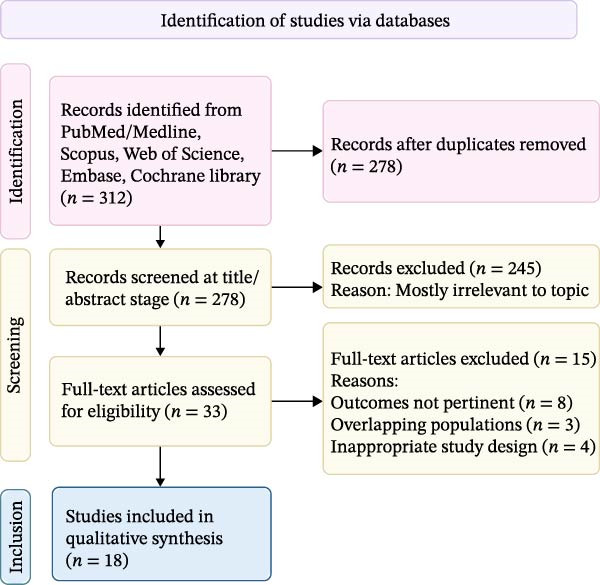
PRISMA‐ScR flow diagram illustrating the study selection process.

The study selection process is illustrated in Figure 1. Of 312 records initially retrieved, 278 remained after duplicate removal. Title and abstract screening excluded 245 records. Full‐text review of the remaining 33 articles led to exclusion of 15 studies (irrelevant outcomes, *n* = 8; overlapping populations, *n* = 3; and inappropriate design, *n* = 4). Eighteen studies met the inclusion criteria and were included in the qualitative synthesis.

### 2.5. Data Extraction and Synthesis

Two reviewers independently extracted data using a standardized form. Extracted items included: study characteristics (authors, year, country); study design and sample (type, size, subject, or model characteristics); diagnostic criteria for occlusal trauma and periodontal disease (tooth mobility, CAL, and RBL); exposure methodology (selective grinding, artificial occlusal interference, and mechanical loading devices); outcomes assessed (periodontal parameters, histological and radiographic findings, biomarker expression—including IL‐6, MMPs, and RANKL—gene expression, and enzyme activation levels); and key findings comparing test and control groups.

Data were summarized descriptively and organized into tables stratified by study type (clinical, animal, and in vitro) to facilitate comparison of outcomes and identification of mechanistic themes.

## 3. Results

Eighteen studies met the inclusion criteria and were incorporated into the qualitative synthesis: eight clinical studies, seven animal model investigations, and three in vitro experiments. The methodological approaches and outcome measures varied considerably across study types, reflecting the multifaceted nature of occlusal trauma research.

Clinical evidence (*n* = 8): five observational studies [9–12], one radiographic survey [13], and two ex‐vivo investigations of human teeth/tissues [14, 15] examined the impact of occlusal loading in periodontitis. Most reported greater PD progression and elevated crevicular aMMP‐8 in sites subjected to traumatic contacts. aMMP‐8—interpreted as a marker of inflammatory collagenolysis rather than a trauma‐specific signal—should be co‐interpreted with clinical/radiographic evidence of traumatic contacts.

Animal models (*n* = 7): rodent studies [16–22] applied controlled occlusal overload to ligature‐induced periodontitis, demonstrating amplified bone loss, heightened pro‐inflammatory cytokines, and increased aMMP‐8 expression relative to periodontitis‐only controls consistent with heightened degradative activity under combined mechanical and inflammatory stress.

Importantly, “occlusal overload” lacks a standardized operational definition in the animal literature. The term encompasses heterogeneous experimental interventions, including composite buildups (1 mm height), metal wire interference, occlusal elevation, force unloading by antagonist extraction, and dietary manipulation that differ substantially in magnitude, direction, duration, and biomechanical relevance to chronic human occlusal trauma. This methodological heterogeneity should be considered when interpreting the collective findings (see Section 4.7 for detailed discussion).

In vitro/hybrid designs (*n* = 3): two hybrid studies combined animal or human tissues with cell cultures [15, 19], while three used periodontal‐ligament cell lines exclusively [23–25]. Excessive cyclic strain up‐regulated MMP‐8, MAPK, and NF‐κB pathways, corroborating in vivo findings.

Tables 1–3 summarize the key characteristics and principal findings of the included clinical, animal, and in vitro studies, respectively. The tables have been reorganized to highlight the relationships among occlusal factors (cause), therapeutic interventions (treatment), and molecular pathways (mechanism/cascade), as suggested by the reviewers. Broadly, the clinical studies assessed the effects of occlusal trauma in patients with periodontal disease, providing real‐world insights into disease exacerbation under traumatic forces. The animal studies enabled controlled investigation of occlusal overload, frequently through experimental models comparing periodontitis with and without induced trauma thereby isolating its specific pathological effects. The in vitro studies focused on elucidating the cellular and molecular responses, particularly within PDL cells, subjected to excessive mechanical stress. In the sections below, results are categorized by study type, with particular emphasis on findings related to collagen degradation and MMP‐8 activation.

**Table 1:** Characteristics and key findings from human clinical studies investigating the role of occlusal trauma in periodontal disease (reorganized to highlight cause/treatment, mechanism, and key outcomes per reviewer 2 suggestion).

Author/year/design	Sample/age	Occlusal trauma criteria (Cause)	Periodontal disease criteria	Intervention/treatment	Key mechanisms/markers	Conclusion
Inchingolo et al. [9] (2021) Clinical interventional	*n* = 15 Humans 41–62 years	Pre‐existing trauma: tooth mobility, fremitus, occlusal discrepancies	Moderate–severe periodontitis, PD >4 mm, BOP	Periodontal therapy ± occlusal adjustment/splinting	Subgingival pathogen reduction (*T. forsythia*, *T. denticola*)	SRP + occlusal adjustment is more effective than SRP alone. Synergistic effect reducing PD, BoP, and subgingival pathogens up to 80%
Meynardi et al. [10] (2016) Observational clinical	*n* = 260 patients Group 1: 81 Group 2:179	40‐μm articulating paper (blue = static, red = dynamic occlusion)	PD, BOP, plaque samples for bacterial monitoring	Dental cleaning ± occlusal adjustment	Microbial profile shift (coccus content)	Occlusal adjustment improved periodontal health by reducing tissue stress. Group 2 maintained 70%–77% coccus content vs. 26%–53% in Group 1
Ríos et al. [11] (2021) Retrospective case–control	*n* = 372 records 167 cases 205 controls Cases: 50.6 years Controls: 45.8 years	≥1 tooth with widened PDL space, tooth mobility/fremitus, occlusal discrepancies	Cases: ≥2 nonadjacent sites with CAL loss, PD ≥4 mm, BOP Controls: PD ≤3 mm, minimal BOP	Cross‐sectional analysis (no intervention)	Association analysis	Occlusal trauma is significantly associated with periodontitis, suggesting a role as a contributing factor
Nalini et al. [26] (2024) Clinical trial	*n* = 40 humans 21–44 years	Clinical tooth mobility, radiographically widened PDL space, tooth wear with thickened lamina dura	PD ≥5 mm, CAL ≥3 mm, gingival index	Group I: SRP + T‐scan calibration Group II: SRP + T‐scan recording only	Force distribution normalization	T‐Scan occlusal calibration improved PPD, CAL, and force distribution in periodontitis patients with occlusal trauma
Venugopalan et al. [12] (2024) Randomized controlled clinical trial	*n* = 30 patients Group I: 14 patients, 262 teeth Group II: 16 patients, 276 teeth	Pre‐existing high occlusal forces assessed using T‐scan	PD, CAL, and teeth mobility	Group I: flap surgery Group II: flap surgery + coronoplasty	Mobility reduction without CAL/PPD change	Occlusal adjustment with flap surgery significantly reduces tooth mobility but does not substantially improve CAL or PPD
Luchian et al. [14] (2019) Observational study	*n* = 66 human teeth (22 premolars, 44 molars); age not specified	Pre‐existing occlusal conditions. Presence/absence of antagonist contact	CAL ≥4 mm, Grade II mobility, BOP radiographic bone loss (Group 1:1–2/3; Group 2: >2/3 alveolar height)	Cross‐sectional analysis of root resorption	Root resorption quantification	Severe periodontal disease with antagonist contact is associated with a significant increase in root resorption. Group 2 with antagonist contact had the highest resorption
Tagger‐Green et al. [13] (2023) Observational study	*n* = 1950 full‐mouth radiographic surveys; age not specified	Occlusal/incisal tooth wear, widening of PDL space	Interproximal marginal bone loss quantified relative to root length	Cross‐sectional radiographic analysis	PDL widening, bone loss correlation	Tooth wear positively correlated with periodontal space widening (OR = 2.585) and marginal bone loss (OR = 2.767)

*Note:* This table summarizes selected clinical studies evaluating the impact of occlusal trauma on periodontal disease. Columns have been reorganized to highlight cause (occlusal trauma criteria), treatment (intervention), and key mechanisms/markers.

Abbreviations: BOP, bleeding on probing; CAL, clinical attachment level; GI, gingival index; PD, probing depth; PDL, periodontal ligament.; PPD, probing pocket depth; SRP, scaling and root planing

**Table 2:** Characteristics and key findings from animal models investigating the role of occlusal trauma in periodontal disease (reorganized per reviewer 2).

Author/year/design	Animal model/*n*	Occlusal trauma induction (cause)	Periodontitis model	Intervention/treatment	Key mechanisms/cascade	Conclusion
Wei et al. [15] (2021) Case control + mouse model	*n* = 18 patients + 80 BALB/c mice 4–5 weeks old	Composite resin (~1 mm height) adhered to molars, creating premature contact	*P. gingivalis* suspension application	YAP inhibitor (XAV939) treatment	YAP/JNK pathway IL‐6, TNF‐α upregulation	Occlusal trauma worsens bone loss via YAP/JNK‐mediated inflammation. XAV939 treatment reduced bone loss and cytokine expression
Liu et al. [17] (2025) Experimental animal	*n* = 6, 84 C57BL mice	Occlusal trauma applied to a subset of mice	Periodontitis induced	Molecular analysis of fibrin degradation pathways	Plasminogen/plasmin system suppression: fibrin deposition	Occlusal trauma exacerbates periodontitis by promoting excessive fibrin deposition through suppression of the plasminogen/plasmin system
Jiang et al. [16] (2022) Experimental animal	*n* = 25–30 male SD rats 7‐week‐old, 250 ± 20 g	Metal wire interference	qRT‐PCR confirmed (NLRP3 and IL‐1β)	Five groups: control, trauma, periodontitis, trauma + periodontitis, + NLRP3 inhibitor (glyburide)	NLRP3‐dependent pyroptosis; osteoclast activation	Occlusal trauma + periodontitis exacerbates pyroptosis and inflammation, leading to severe bone loss. NLRP3 inhibition mitigated the effects
Nakatsu et al. [18] (2014) Experimental animal	*n* = 60 male Lewis rats, 9‐week‐old, LPS‐Immunized (48) and nonimmunized (12)	Metal wire interference assessed via attachment loss and collagen fiber damage	*E. coli* LPS application	Four groups: trauma, Inflammation, trauma + inflammation, PBS control	Immune complex formation, osteoclast activity increases	Occlusal trauma + periodontal inflammation significantly increases attachment loss, osteoclast activity, and immune complex formation
Xu et al. [21] (2020) Experimental animal	*n* = 18 male Wistar rats 6‐week‐old, ~200 g	1 mm orthodontic wire bonded to the occlusal surface using photopolymerized resin	Not explicitly described	Three groups: control, occlusal trauma, trauma + IKK‐2 inhibitor	IKK‐NF‐κB signaling Osteogenesis suppression	Occlusal trauma exacerbates bone loss through IKK‐NF‐κB‐mediated inflammation. IKK‐2 inhibitors partially mitigated effects by enhancing osteogenesis
Pan et al. [19] (2019) Experimental animal	*n* = 20 male BALB/c mice 4–5 weeks old	Traumatic occlusion assessed via micro‐CT for alveolar bone resorption	*P. gingivalis* application with micro‐CT, histopathology, and molecular analyses	Four groups: control, traumatic occlusion, P.g, traumatic occlusion + P.g	IKK‐NF‐κB pathway Hippo‐YAP pathway	Traumatic force and LPS activated the IKK‐NF‐κB pathway. The combined group showed the most severe bone resorption with the widest PDL and significant BV/TV reduction
Soenjaya et al. [20] (2015) Experimental study	Multiple Bsp^−/−^ and wild‐type mice 3–20 weeks	Naturally occurring malocclusion in Bsp^−/−^ mice, exacerbated by a hard diet	Spontaneous periodontal defects due to genetic knockout of bone sialoprotein	Comparison of soft diet vs. complex diet effects on periodontal breakdown	Genetic susceptibility (Bsp^−/−^) cementum loss, root resorption	Excessive occlusal forces exacerbate periodontal defects in Bsp^−/−^ mice, suggesting occlusal management may mitigate pathology in susceptible patients
Zhu et al. [22] (2022) Experimental, controlled in vivo	*n* = 60 mice, Male C57BL/6 wild‐type, 6 weeks old	Occlusal force unloading by the extraction of the left maxillary first molar	Ligature‐induced: 5–0 silk ligature around the mandibular left first molar	Five groups: Control, PD, PD + EX, EX, plus long‐term controls (Con‐12 and EX‐12)	M1/M2 macrophage polarization ratio Osteoclastogenesis	Occlusal force unloading exacerbated alveolar bone loss and osteoclastogenesis in ligature‐induced periodontitis by increasing the M1/M2 macrophage polarization ratio

*Note:* This table summarizes preclinical studies evaluating the impact of occlusal trauma on periodontal disease. A new column, “Key Mechanisms/Cascade,” has been added to highlight the molecular pathways identified in each study. NF‐κB, nuclear factor kappa‐light‐chain‐enhancer of activated B cells.

Abbreviations: BV/TV, bone volume to total volume ratio; IKK, IκB kinase; IL, interleukin; LPS, lipopolysaccharide; MMP, matrix metalloproteinase; P.g., *Porphyromonas gingivalis*; PD, probing depth; PDL, periodontal ligament; RANKL, receptor activator of nuclear factor κB ligand; TNF‐α, tumor necrosis factor‐alpha; TRAP, tartrate‐resistant acid phosphatase; YAP, yes‐associated protein.

**Table 3:** Characteristics and main findings from selected In vitro studies (reorganized per reviewer 2).

Author/year	Cell/tissue type	Force applied (cause)	Parameters assessed	Key mechanisms/cascade	Findings	Conclusion
Wei et al. [15](2021)	L929 mouse fibroblast cell line	Cyclic compressive stress (0.5 Hz, 0.5 mm depth, 2 h) using a four‐point bending device	YAP dephosphorylation, nuclear transfer, inflammatory factors (qRT‐PCR), JNK pathway activity	YAP/JNK/AP‐1 pathway IL‐6, TNF‐α cascade	Combined cyclic stress + LPS increased YAP dephosphorylation, nuclear pJNK, and inflammatory cytokines (Ap‐1, Il6, Tnfα), reduced by XAV939	YAP‐targeted therapies could reduce inflammation and tissue destruction in periodontitis with occlusal trauma
Tantilertanant et al. [23] (2019)	Primary human PDL cells	Cyclic tensile force (10% elongation, 60 rpm, 2–6 h)	Gene (IL6, MMPs, TIMPs, and IL6R) and protein (IL6 and MMP3) expression via qPCR and ELISA	IL‐6/IL‐6R signaling PI3K/MAPK cascade MMP‐3 upregulation	Force upregulated IL6 and MMPs with sustained MMP3 protein at 48 h. Anti‐IL6 antibodies significantly reduced MMP3. IL6‐induced MMP3 via PI3K and MAPKs, not JAK/STAT3	Mechanistic links between occlusal trauma and periodontal pathology; IL6 and PI3K/MAPK as potential therapeutic targets
Xu et al. [21] (2020)	MC3T3‐E1 pre‐osteoblast cells (mouse)	Cyclic uniaxial compressive stress (4000 μstrain, 0.5 Hz) ± *P. gingivalis* LPS ± IKK‐2 inhibitor VI	p65, p‐IκBα, osteoblast markers (Osterix, ALP, BSP, OCN), ALP activity, Wnt/β‐catenin signaling	IKK‐NF‐κB activation, Wnt/β‐catenin suppression, osteogenic inhibition	Force + Pg. LPS inhibited osteogenic differentiation by activating IKK‐NF‐κB, suppressing Wnt/β‐catenin, and osteogenic markers. IKK‐NF‐κB blocking restored osteogenesis	Traumatic force + LPS inhibit osteogenesis via IKK‐NF‐κB activation, reversible by IKK‐NF‐κB inhibitors
Pan et al. [19] (2019)	Mouse fibroblast L929 cells and pre‐osteoblasts	Mechanical force: CCS and cyclic tensile stress (CTS) via force device	qPCR: YAP, JNK/AP‐1, inflammatory cytokines. Immunofluorescence: YAP and JNK pathway activation	IKK‐NF‐κB pathway Wnt/β‐catenin suppression osteoblast marker reduction	Force + LPS activated IKK‐NF‐κB, reducing osteoblast markers and ALP activity, inhibiting Wnt/β‐catenin. IKK‐NF‐κB inhibition rescued osteoblast markers	Traumatic force and LPS activate IKK‐NF‐κB signaling, reducing osteoblast markers and inhibiting Wnt/β‐catenin signaling. IKK‐NF‐κB inhibition rescued bone formation
Zhao et al. [25] (2022)	Human periodontal ligament stromal cells (hPDLSCs) from 5 healthy donors	Cyclic tensile strain (6% or 12% elongation at 0.1 Hz for 6–24 h)	Gene: IL‐6, IL‐8, VCAM‐1, ICAM‐1 (qPCR). Protein: IL‐6, IL‐8 (ELISA). Surface: VCAM‐1, ICAM‐1 (immunostaining and flow cytometry)	TNF‐α modulation IL‐6/IL‐8 regulation Cell adhesion molecules	Cyclic strain modulated TNF‐α‐induced inflammatory responses, suppressing IL‐6 and VCAM‐1 but enhancing IL‐8 protein levels	Cyclic tensile strain has anti‐inflammatory and pro‐inflammatory effects depending on the mediator and force magnitude
Xu et al. [24] (2022)	MC3T3‐E1 osteoblastic cells (mouse)	4000 μstrain, 0.5 Hz cyclic compressive force for 6 h ± Pg. LPS ± rWnt4 ± ROCK inhibitor	mRNA/protein levels (ALP, Runx2, Wnt4, RANKL, RhoA, and p65) via qPCR and Western blot	IKK‐NF‐κB pathway, ROCK signaling, Wnt4 rescue mechanism	Force + LPS suppressed ALP, Runx2, Wnt4; activated IKK‐NF‐κB; rWnt4 reversed suppression, promoted osteogenesis, suppressed p65/RANKL	Traumatic force + Pg. LPS synergistically inhibits osteogenesis via IKK‐NF‐κB and ROCK pathways; rWnt4 can rescue bone formation
Zhu et al. [22] (2022)	THP‐1‐derived macrophages (human cell line)	1 g/cm^2^ continuous compressive force for 24 h ± LPS (100 ng/mL)	Gene expression (RT‐qPCR), macrophage polarization (immunofluorescence), cell migration (wound healing assay)	M1/M2 macrophage polarization, cell migration enhancement	Unloading of compressive force under inflammatory conditions (LPS) increased the M1/M2 macrophage polarization ratio and enhanced macrophage migration	Mechanical unloading under inflammatory conditions promotes pro‐inflammatory macrophage polarization and migration, potentially exacerbating inflammation

*Note:* This table summarizes in vitro studies investigating the effects of mechanical forces on periodontal cells. The “Key Mechanisms/Cascade” column has been added to clearly identify the molecular pathways studied in each investigation. NF‐κB:, nuclear factor kappa‐light‐chain‐enhancer of activated B cells

Abbreviations: ALP, alkaline phosphatase; CCS, cyclic compressive stress; CTS, cyclic tensile stress; IKK, IκB kinase; IL, interleukin; JNK, c‐Jun N‐terminal kinase; LPS, lipopolysaccharide; MMP, matrix metalloproteinase; PDL, periodontal ligament; RANKL, receptor activator of nuclear factor κB ligand; TIMP, tissue inhibitor of metalloproteinases; TNF‐α, tumor necrosis factor‐alpha; YAP, yes‐associated protein.

Animal studies have used heterogeneous methods to modify occlusal loading, including occlusal interference, force unloading, and dietary manipulation (Table 4), limiting direct comparison and clinical extrapolation.

**Table 4:** Experimental paradigms for inducing occlusal overload in animal models.

Method	Magnitude	Duration	Clinical relevance	Studies
Composite buildup	approximately 1 mm height; supraphysiological	Acute (days–weeks)	Limited; exceeds chronic human trauma	Wei et al. [15] Pan et al. [19]
Metal wire interference	Variable; moderate–high	Acute–subacute	Moderate; simulates premature contacts	Jiang et al. [16] Nakatsu et al. [18]
Force unloading (extraction)	Complete removal	Chronic	Limited explores the opposite extreme	Zhu et al. [22]
Dietary manipulation	Indirect; variable	Chronic	Moderate; reflects natural variation	Soenjaya et al. [20]

### 3.1. Human Clinical Studies

Six clinical studies published between 2016 and 2024 evaluated the impact of occlusal trauma on periodontal parameters. These studies varied in design, sample size (ranging from 20 to 372 participants), diagnostic criteria for trauma and periodontitis, and treatment protocols. Diagnostic approaches for occlusal trauma included clinical assessment of tooth mobility and fremitus, articulating paper analysis, T‐scan digital occlusal analysis, and radiographic evaluation of PDL widening, reflecting the absence of standardized diagnostic criteria in this field (Table 5).

**Table 5:** Diagnostic approaches for occlusal trauma in clinical studies.

Diagnostic method	Parameters assessed	Advantages	Limitations
Clinical mobility assessment	Tooth mobility grades (Miller classification)	Simple, no equipment needed, widely used	Subjective, inter‐examiner variability
Fremitus detection	Vibration during function	Functional assessment, no equipment	Subjective, requires patient cooperation
Articulating paper	Contact location, static/dynamic contacts	Inexpensive, widely available	Qualitative only, no force measurement
T‐scan digital analysis	Force magnitude, timing, distribution	Quantitative, reproducible, time‐sequenced data	Equipment cost, technique sensitivity, and interpretation variability
Radiographic PDL widening	PDL space width, lamina dura changes	Objective measurement documents chronicity	2D limitation, projection variability, late sign

Several studies demonstrated that combining periodontal therapy with occlusal adjustment or calibration led to significantly greater improvements in clinical outcomes compared to periodontal therapy alone [9, 10, 26]. Combined nonsurgical periodontal therapy and occlusal adjustment produced superior results: PPD 0.8 ± 0.2 mm vs. 0.3 ± 0.2 mm (*p*  < 0.01) [9,10,12]; BOP 18% vs. 7% (*p*  < 0.05) [9, 10, 26]; mobility grade decreased 0.6 ± 0.3 (*p*  < 0.05) [10]. However, it should be noted that these effect sizes, while statistically significant, represent modest absolute improvements that may not exceed the minimal clinically important difference for all patients.

Meynardi et al. [10] reported a significant reduction in periodontal pathogens, including *Tannerella forsythia* and *Treponema denticola*, following occlusal adjustment. High occlusal forces were positively associated with greater disease severity, as evidenced by increased CAL, PPD, tooth mobility, and RBL [11, 12].

Notably, clinical outcomes were not uniformly positive across all parameters. Venugopalan et al. [12] found that occlusal adjustment with flap surgery significantly reduced tooth mobility but did not demonstrate substantial improvements in CAL or PPD. This discordance suggests that the therapeutic effects of occlusal intervention may be context‐dependent, with mobility reduction (reflecting biomechanical stabilization) potentially dissociated from attachment‐level outcomes (reflecting periodontal regeneration).

### 3.2. Radiographic and Tooth‐Based Observations in Humans

Observational analyses of extracted teeth and radiographic surveys provided compelling evidence linking occlusal factors to periodontal breakdown. In a microscopic study, Luchian et al. [14] found that teeth with severe alveolar bone loss (> two‐thirds of normal height) exhibited significantly greater root surface and volume resorption than those with moderate alveolar bone loss (*p* = 0.024 and *p* = 0.003, respectively). The presence of antagonist contacts further exacerbated resorptive changes.

Similarly, Tagger‐Green et al. [13] reported that tooth wear was significantly associated with widening of the PDL space (OR = 2.585) and marginal bone loss (OR = 2.767) in a retrospective survey of 1950 full‐mouth radiographs. These findings highlight that occlusal trauma‐related factors, including tooth wear and contact stress, are closely linked to periodontal tissue deterioration and root resorption.

### 3.3. In Vivo Experimental Animal Studies

Seven rodent studies investigated the effects of occlusal trauma under controlled experimental conditions. In most studies, experimental periodontitis was induced (e.g., via ligatures or *Porphyromonas gingivalis* inoculation) followed by application of occlusal overload (typically by cementing a composite resin buildup on a molar to create a premature contact). As noted above, these experimental paradigms differ substantially from chronic human occlusal trauma in terms of force magnitude, duration, and biomechanical characteristics (Table 4).

Across these models, occlusal trauma consistently exacerbated inflammation, bone loss, and matrix degradation, particularly in conjunction with microbial challenge. The key findings can be summarized as follows.

#### 3.3.1. Amplified Tissue Destruction

Controlled overload consistently amplified alveolar bone loss (e.g., μCT BV/TV↓ and CEJ–ABC distance↑) and attachment loss relative to ligature‐only or periodontitis‐only controls [16–19, 21].

#### 3.3.2. Key Signaling Pathways

Multiple studies converged on the IKK–NF‐κB signaling axis as a central mediator of occlusal trauma‐induced damage. Pharmacologic IKK‐2 blockade partially rescued osteogenesis and limited bone resorption [19, 21]. Additionally, the NOD‐like receptor family pyrin domain containing 3 (NLRP3) inflammasome pathway was implicated in pyroptosis‐mediated bone loss, with NLRP3 inhibition (glyburide) attenuating pyroptotic markers and tissue destruction [16].

#### 3.3.3. Specific Mechanistic Findings

Liu et al. [17] reported that trauma aggravated periodontal destruction by impairing fibrin degradation through suppression of the plasminogen/plasmin system. Nakatsu et al. [18] found that trauma combined with LPS‐induced inflammation produced greater attachment loss and immune complex deposition than either factor alone. Pan et al. [19] confirmed that traumatic occlusion combined with *P. gingivalis* infection led to the most severe bone resorption among experimental groups.

#### 3.3.4. Host Susceptibility Factors

In a genetic model, Bsp^−^/^−^ mice subjected to occlusal overload developed pronounced periodontal defects, including loss of cementum and root resorption [20], underscoring the role of host factors in modulating the response to mechanical stress.

#### 3.3.5. Bidirectional Effects of Mechanical Loading

Interestingly, Zhu et al. [22] showed that occlusal unloading (by antagonist extraction) paradoxically worsened alveolar bone loss in ligature‐induced periodontitis models by skewing M1/M2 macrophage polarization. This finding suggests that both excessive and insufficient mechanical loading can disrupt periodontal homeostasis, highlighting the importance of physiologic loading for tissue maintenance.

#### 3.3.6. Translational Considerations

While these animal studies provide valuable mechanistic insights, the acute, supraphysiological loading conditions employed (e.g., 1 mm composite buildups creating immediate premature contacts) may not reflect the chronic, variable‐magnitude forces characteristic of human occlusal trauma. These findings establish biological plausibility for the role of mechanical factors in periodontal pathology but should be interpreted cautiously when extrapolating to clinical decision‐making.

### 3.4. In Vitro Studies

The in vitro studies provided crucial mechanistic insights into how mechanical forces associated with occlusal trauma modulate cellular and molecular responses within periodontal tissues.

#### 3.4.1. YAP Signaling Pathway

Wei et al. [15] demonstrated that cyclic compressive stress applied to L929 mouse fibroblasts, particularly when combined with lipopolysaccharide (LPS) stimulation, led to increased dephosphorylation of YAP, activation of the JNK pro‐inflammatory pathway, and upregulation of cytokines such as AP‐1, IL‐6, and TNF‐α. These effects were significantly attenuated by YAP inhibition (XAV939), suggesting that YAP‐targeted interventions may help mitigate inflammation and tissue destruction.

#### 3.4.2. IL‐6/MMP Axis

Tantilertanant et al. [23] observed that cyclic tensile force applied to primary human PDL cells resulted in elevated expression of IL‐6 and MMPs, including sustained MMP‐3 protein expression and upregulation of IL‐6 receptor (IL‐6R). Inhibition of IL‐6 signaling significantly reduced MMP‐3 levels, suggesting that IL‐6 and the downstream PI3K/MAPK signaling cascade are key mediators of the catabolic response to mechanical overload.

#### 3.4.3. Force‐Dependent Inflammatory Modulation

Zhao et al. [25] examined the effect of cyclic tensile strain on human PDL stromal cells (hPDLSCs) exposed to TNF‐α. Mechanical loading suppressed IL‐6 and vascular cell adhesion molecule‐1 (VCAM‐1) expression while enhancing IL‐8 production, indicating a force‐ and mediator‐dependent modulation of inflammatory signaling. This finding highlights the complexity of mechanotransduction, in which different force parameters can elicit distinct—and sometimes opposing—cellular responses.

#### 3.4.4. IKK‐NF‐κB Pathway and Osteogenic Suppression

Multiple in vitro studies converged on the IKK‐NF‐κB signaling pathway as a central mediator of mechanical stress‐induced effects on bone metabolism. Xu et al. [21] reported that traumatic compressive forces, particularly in the presence of *P. gingivalis* LPS, inhibited osteogenic differentiation in MC3T3‐E1 pre‐osteoblasts by activating IKK‐NF‐κB, which downregulated Wnt/β‐catenin signaling and osteogenic markers. Inhibition of IKK‐NF‐κB reversed these effects. In a subsequent study, Xu et al. [24] demonstrated that traumatic force combined with LPS activated both IKK‐NF‐κB and ROCK signaling pathways; treatment with recombinant Wnt4 reversed these suppressive effects and promoted osteogenesis. Pan et al. [19] corroborated these findings, showing that both compressive and tensile forces activated IKK‐NF‐κB signaling in mouse fibroblasts and pre‐osteoblasts, resulting in downregulation of osteoblast differentiation markers.

#### 3.4.5. Macrophage Polarization

Zhu et al. [22] found that mechanical unloading of THP‐1‐derived macrophages under inflammatory conditions enhanced M1 polarization and increased cell migration, suggesting that both excessive and insufficient mechanical stimuli can exacerbate inflammatory responses.

Collectively, these in vitro studies highlight that mechanical stress associated with occlusal trauma can amplify inflammatory and catabolic pathways, particularly when compounded by bacterial challenge. The convergence of multiple studies on the IKK‐NF‐κB pathway suggests this may represent a promising therapeutic target, though validation in human studies is required before clinical translation. Targeting specific molecular mediators, such as YAP, IL‐6, NF‐κB, and Wnt signaling, may offer strategies to attenuate the tissue‐destructive effects of pathological mechanical loading in periodontal disease.

## 4. Discussion

This scoping review reinforces the long‐held but debated view that while occlusal trauma is not an initiating cause of periodontitis, it can substantially exacerbate disease severity when combined with plaque‐induced inflammation. Since the seminal works of Glickman and Smulow [27] and Waerhaug [28], the role of traumatic occlusal forces in attachment loss has been a topic of contention. However, findings from clinical, animal, and cellular studies over the past decade provide compelling evidence of a modifying role for occlusal trauma in periodontal disease pathogenesis.

### 4.1. Occlusal Trauma and the Subgingival Microbiota

Emerging data suggest that occlusal trauma may modulate the subgingival microbial environment, fostering dysbiosis. Clinical studies have demonstrated that periodontal therapy combined with occlusal adjustment not only improves clinical outcomes but also significantly reduces pathogenic bacteria, including *T. forsythia* and *T. denticola* [9, 10]. Meynardi et al. [10] further noted that patients who received occlusal adjustment exhibited a more stable and favorable microbial profile over time than those who received scaling alone.

Importantly, these findings should not be interpreted to suggest that antimicrobial therapy alone could substitute for addressing occlusal trauma. The observed microbial shifts likely reflect improved tissue healing and reduced PD following combined therapy, rather than a direct antimicrobial effect of occlusal adjustment. Occlusal trauma appears to perpetuate a dysbiotic environment by sustaining tissue injury and inflammation; thus, antimicrobial approaches without concurrent biomechanical correction are unlikely to achieve durable microbial homeostasis [9]. This interpretation aligns with current principles of multifactorial disease management and antibiotic stewardship, which emphasize addressing root causes rather than relying solely on antimicrobial suppression.

### 4.2. Therapeutic Sequence and Impact on Clinical Parameters

The timing and integration of occlusal adjustment in periodontal therapy appear to influence both clinical outcomes and biomarker levels. Earlier studies by Hakkarainen et al. [2] demonstrated that occlusal adjustment of hypermobile teeth significantly reduced total protein and aMMP‐8 levels in sulcular fluid, with effects amplified by SRP. Such decreases likely reflect reduced overall inflammatory collagenolysis and should not be taken as evidence that aMMP‐8 is specific to traumatic loading. Multiple studies have confirmed that SRP, when coupled with occlusal calibration, yields superior reductions in PD, BoP, and tooth mobility compared with SRP alone [9, 26].

However, the clinical evidence is not uniformly positive. Venugopalan et al. [12] found that occlusal adjustment significantly reduced tooth mobility but did not improve CAL or PPD. This discordance between mobility reduction and attachment‐level outcomes suggests that occlusal adjustment may primarily yield biomechanical stabilization rather than periodontal regeneration. Mobility improvement likely reflects redistribution of occlusal forces and reduced PDL strain, which may not translate into connective tissue regeneration or reversal of attachment loss [29]. This distinction is clinically important: occlusal intervention should be viewed as a supportive measure that may improve functional outcomes and patient comfort, but expectations for regenerative benefits should be tempered. The context‐dependent nature of these effects, including disease severity, healing capacity, and concurrent microbial burden, likely explains the heterogeneity in clinical outcomes across studies.

Furthermore, while statistically significant, the reported improvements in PD (typically 0.5–0.8 mm beyond scaling alone) represent modest effect sizes that may not exceed the minimal clinically important difference for all patients. Combined nonsurgical periodontal therapy and occlusal adjustment produced superior results compared with periodontal therapy alone: PPD 0.8 ± 0.2 mm vs. 0.3 ± 0.2 mm (*p*  < 0.01); BOP 18% vs. 7% (*p*  < 0.05); mobility grade decrease 0.6 ± 0.3 (*p*  < 0.05). These findings suggest that routine occlusal adjustment for all periodontitis patients may not be warranted; instead, selective intervention in patients with documented occlusal discrepancies and progressive disease may optimize the benefit‐to‐burden ratio.

### 4.3. Occlusal Trauma as a Co‐Factor in Periodontal Disease

Occlusal trauma has been consistently recognized as a significant co‐factor that is associated with exacerbated progression and severity of periodontal disease. Retrospective and observational clinical studies have demonstrated that patients presenting with both periodontitis and signs of occlusal trauma exhibit significantly greater CAL, increased tooth mobility, and more pronounced radiographic alveolar bone loss compared to individuals with periodontitis alone [11, 12].

The relationship between occlusal trauma and periodontitis is likely bidirectional. While excessive occlusal forces may exacerbate tissue destruction in the presence of inflammation (forward causation), periodontal breakdown itself may predispose to traumatic occlusal forces through pathologic tooth migration, altered contact relationships, and reduced periodontal support (reverse causation) [13, 14, 30]. This bidirectional interplay underscores the importance of concurrent management of both microbial and mechanical factors, while recognizing that occlusal trauma functions as a modifier rather than an initiating cause of periodontal disease.

### 4.4. Mechanistic Insights: Inflammation and Matrix Degradation

**Figure 2:**
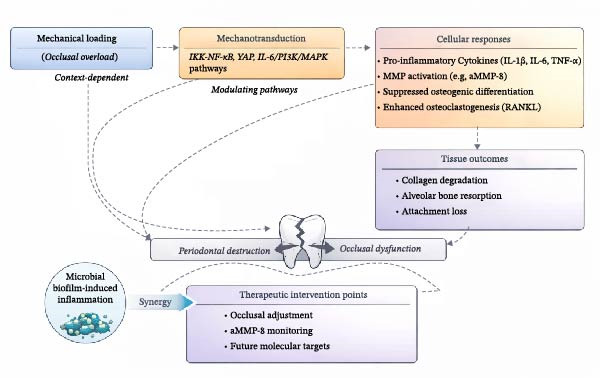
Conceptual framework: occlusal trauma as a modifying factor in periodontitis.

Mechanistic insights should be interpreted as evidence of biological plausibility rather than clinical behavior, as experimental systems isolate specific force‐responsive pathways. Mechanical stress (cyclic stretch, compression, and tensile loading) activates pro‐inflammatory and catabolic pathways in PDL cells, fibroblasts, and osteoblasts, with central mediators, including YAP, IL‐6, and IKK‐NF‐κB. These pathways currently represent theoretical targets; their clinical modulation is experimental and not validated in humans. As illustrated in Figure 2, altered occlusal loading may exacerbate periodontal inflammation by modulating host immune responses, mechanotransduction pathways, and proteolytic activity in the context of microbial dysbiosis.

Animal and in vitro models show occlusal trauma exacerbates local inflammation, enhances fibrin deposition, and suppresses osteogenic activity via NLRP3, Wnt/β‐catenin, and NF‐κB signaling [17]. Notably, several studies have demonstrated that these mechanotransductive pathways not only amplify pro‐inflammatory cytokine expression but also suppress osteogenic differentiation and bone‐forming activity. For instance, studies revealed that mechanical overload in combination with bacterial challenge inhibited osteogenic markers and Wnt/β‐catenin signaling, establishing a mechanistic link between occlusal forces and bone loss [16, 19, 21]. This imbalance initiates a self‐perpetuating cycle of chronic inflammation, collagen degradation, and structural weakening of the periodontium.

Tantilertanant et al. [23] further demonstrated that cyclic tensile force stimulates IL‐6‐mediated MMP‐3 expression in human PDL cells, reinforcing the catabolic effect of mechanical loading. In parallel, findings by Xu et al. [21, 24] and Pan et al. [19] highlighted the suppression of osteogenesis and the disruption of anabolic signaling pathways, particularly under combined mechanical and microbial stress. Collectively, these findings underscore a complex interplay between mechanical forces and host immune responses, wherein occlusal trauma synergizes with bacterial inflammation to drive extracellular matrix degradation, inhibit bone regeneration, and accelerate periodontal tissue destruction. However, extrapolation of these findings to clinical decision‐making should be cautious, as the experimental forces applied differ substantially from those in human occlusion.

### 4.5. aMMP‐8: Clarification of Proposed Roles and Limitations

aMMP‐8 has emerged as a promising biomarker in periodontal diagnostics, yet its role remains unclear. aMMP‐8 should be understood as a marker of active collagen degradation rather than a specific indicator of occlusal trauma. The mechanistic cascade linking mechanical stress to MMP‐8 activation proceeds as follows: mechanical loading triggers mechanotransduction pathways (including NF‐κB and IL‐6 signaling), which promote neutrophil recruitment and activation, leading to MMP‐8 release and subsequent activation to its collagenolytically active form [8].

However, elevated aMMP‐8 levels may result from any inflammatory process affecting periodontal tissues, including microbial challenge, systemic inflammation, or mechanical stress. In the context of occlusal trauma, aMMP‐8 may serve as an adjunctive tool to identify sites with ongoing matrix breakdown, but it cannot distinguish between occlusal and nonocclusal etiologies [31]. Clinicians should interpret aMMP‐8 results in conjunction with clinical and radiographic findings, recognizing that false positives may occur in the presence of generalized periodontal inflammation. In clinical practice, aMMP‐8 point‐of‐care testing may be most useful for: (1) identifying sites with active matrix degradation that warrant closer monitoring; (2) assessing response to combined periodontal and occlusal therapy; and (3) risk stratification in patients with multiple sites of concern [32]. However, clinicians should not rely on aMMP‐8 alone to diagnose occlusal trauma. Integration with clinical examination (mobility, fremitus, and wear facets) and radiographic findings (PDL widening and bone loss patterns) remains essential for accurate diagnosis.

### 4.6. Methodological Heterogeneity in Animal Models

A significant limitation of the preclinical evidence is the heterogeneity of methods used to induce or assess occlusal overload. The included animal studies employed diverse experimental paradigms, including composite buildups, metal wire interference, occlusal elevation, force unloading, and dietary manipulation, which differ substantially in the magnitude, direction, duration, and biomechanical relevance of the applied forces [1, 12, 18]. This heterogeneity limits the interpretability and reproducibility of findings across studies, as different loading conditions may activate distinct biological responses. For instance, composite buildups creating 1 mm premature contacts represent acute, supraphysiological loading that may not reflect the chronic, variable‐magnitude forces characteristic of human occlusal trauma. Similarly, force unloading by antagonist extraction explores the opposite end of the mechanical spectrum, yet both conditions resulted in tissue damage, suggesting that deviations from physiologic loading in either direction may be detrimental [33]. Future studies should strive for greater standardization of experimental protocols to enable meaningful cross‐study comparisons.

### 4.7. Translational Considerations

While animal and in vitro studies provide valuable mechanistic insights, clinicians should interpret these findings cautiously. The acute, high‐magnitude forces applied in rodent models represent supraphysiological conditions that differ substantially from the chronic, variable‐magnitude forces characteristic of human occlusal trauma. These preclinical findings establish biological plausibility but cannot directly inform clinical decision‐making without validation in human longitudinal studies [1]. Furthermore, while preclinical studies have identified several promising molecular targets, including IL‐6, IKK‐NF‐κB, and YAP signaling pathways, these remain theoretical targets that require validation in human clinical trials before clinical implementation. At present, the only evidence‐based approach to modulating mechanical stress remains clinical occlusal management. Statements implying imminent pharmacologic modulation of these pathways in periodontal practice would be premature without supporting human trials [34]. Future translational research should prioritize human studies to bridge the gap between mechanistic insights and clinical application.

### 4.8. Diagnostic Heterogeneity and Clinical Implications

The included clinical studies employed heterogeneous diagnostic criteria for occlusal trauma, including clinical assessment of tooth mobility and fremitus, articulating paper analysis, T‐Scan digital occlusal analysis, and radiographic evaluation of PDL widening. This diagnostic heterogeneity may contribute to inconsistent clinical outcomes across studies, as different methods capture different aspects of occlusal dysfunction with varying sensitivity and specificity [35]. Digital occlusal analysis offers quantitative force and timing measurements that may improve objectivity compared to traditional methods. However, it should be recognized that digital analysis shifts variability to another measurement domain rather than eliminating it; factors—such as sensor positioning, bite force variation, and interpretation thresholds—introduce new sources of measurement error [36]. The development of standardized diagnostic criteria incorporating multiple assessment modalities would enhance the comparability of future research and facilitate clinical guideline development.

### 4.9. Limitations

This scoping review has several limitations that must be acknowledged. The included studies exhibited considerable variability in study design, diagnostic criteria for occlusal trauma, and therapeutic protocols, resulting in significant heterogeneity that limits the ability to perform direct comparisons or pooled analyses. Many clinical investigations were constrained by small sample sizes, short follow‐up durations, and potential confounding factors, which may affect the robustness and generalizability of the findings. Moreover, much of the mechanistic evidence was derived from in vitro and animal studies, which, despite offering valuable insights into biological pathways, may not fully capture the complex, chronic, and multifactorial nature of human periodontitis. The acute, supraphysiological loading conditions used in many animal models may not reflect chronic human occlusal trauma, limiting translational applicability. The absence of standardized definitions and diagnostic tools for occlusal trauma further complicates cross‐study interpretation and may contribute to inconsistencies in outcomes. Additionally, publication bias cannot be ruled out.

## 5. Conclusions

This scoping review examines the potential role of occlusal trauma in the pathogenesis and progression of periodontal disease. Evidence derived from clinical, animal, and in vitro studies suggests that occlusal trauma may exacerbate periodontal tissue destruction by activating pro‐inflammatory and matrix‐degrading pathways. However, the modest effect sizes observed in clinical studies (typically 0.5–0.8 mm additional PPD reduction) and the heterogeneity of findings suggest that the clinical impact of occlusal intervention may be context‐dependent and limited in scope. While integrating occlusal assessment with adjunctive aMMP‐8 monitoring and targeted therapies may enhance periodontal care, the modest effect sizes observed to date support selective—rather than routine—occlusal intervention; moreover, because current evidence does not show that aMMP‐8 elevations distinguish occlusal from nonocclusal etiologies, results should be corroborated with clinical and imaging findings, and further longitudinal and interventional studies are needed to inform guidelines. Occlusal trauma should be conceptualized as a potential risk modifier rather than a causative factor in periodontal disease. The bidirectional relationship between periodontal destruction and occlusal dysfunction underscores the complexity of this interplay. Adjunctive interventions, such as occlusal adjustment, splinting, or coronoplasty, when combined with conventional periodontal therapy, have been associated with improved clinical outcomes in some studies, including reduced PDs, decreased BoP, and particularly diminished tooth mobility, though attachment‐level improvements have been less consistent.

Mechanistic investigations highlight the involvement of key signaling pathways, such as IL‐6, IKK‐NF‐κB, and YAP, in mediating the potential deleterious effects of mechanical overload, though these remain theoretical therapeutic targets requiring human validation. The role of aMMP‐8 as a biomarker should be understood to reflect active collagen degradation rather than to indicate occlusal trauma per se. Future research should aim to establish standardized diagnostic criteria for occlusal trauma, conduct robust longitudinal and interventional studies with adequate sample sizes and follow‐up durations, and explore targeted molecular therapies. Given the current evidence base, selective rather than routine occlusal intervention—targeting patients with documented occlusal discrepancies and progressive disease—may optimize the benefit‐to‐burden ratio. Such efforts will be crucial for establishing causality, refining treatment strategies, and ultimately enhancing patient outcomes through a more comprehensive approach to periodontal care.

## Author Contributions

Conceptualization: Pietro Leone and Mauno Könönen. Methodology, investigation, writing – original draft preparation: Julie Toby Thomas. Software, resources, data curation: Sukumaran Anil. Validation: Timo Sorsa and Mauno Könönen. Formal analysis, supervision: Pietro Leone. Writing – review and editing, visualization, funding acquisition: Timo Sorsa. Project administration: Mauno Könönen.

## Funding

This research received no external funding.

## Disclosure

All the authors have read and agreed to the published version of the manuscript. All machine‐assisted content was reviewed and verified by the authors.

## Conflicts of Interest

The authors declare no conflicts of interest.

## Data Availability

No new data were generated. All data were obtained from published sources available in public databases (PubMed/MEDLINE, Web of Science, Scopus, Embase, and Cochrane Library) and via the original articles cited.
